# High-Protein Foods for Dysphagia: Manipulation of Mechanical and Microstructural Properties of Whey Protein Gels Using De-Structured Starch and Salts

**DOI:** 10.3390/gels8070399

**Published:** 2022-06-23

**Authors:** Cai Ling Ang, Kelvin Kim Tha Goh, Kaiyang Lim, Lara Matia-Merino

**Affiliations:** 1 School of Food and Advanced Technology, Massey University, Private Bag 11222, Palmerston North 4442, New Zealand; c.ang@massey.ac.nz (C.L.A.); k.t.goh@massey.ac.nz (K.K.T.G.); 2 Riddet Institute, Massey University, Private Bag 11222, Palmerston North 4442, New Zealand; 3 ES-TA Technology Pte Ltd., 21 Jalan Mesin, Singapore 368819, Singapore; kaiyang.lim.john@gmail.com

**Keywords:** whey protein, de-structured starch, food gels, gel product design, gel property analysis, dysphagia, texture, rheology, microstructure, ionic strength

## Abstract

This study focuses on understanding the effect of ionic strength on the mechanical and microstructural properties of novel composite gels containing 13% whey protein isolate (WPI) and 4% de-structured waxy potato starch (DWPS). The DWPS is a physically modified waxy potato starch treated at 140 °C for 30 min under constant shear. Thermodynamic incompatibility between WPI and DWPS was observed upon the addition of NaCl (~75 mM) or CaCl_2_ (10–75 mM). The combined effects of such thermodynamic incompatibility with the changes in protein connectivity induced by varied ionic strength led to the formation of distinctive gel structures (inhomogeneous self-supporting gels with a liquid centre and weak gels with paste-like consistency) that were different from thermodynamic compatible homogeneous self-supporting gels (pure WPI and WPI + maltodextrin gels). At ≥ 250 mM NaCl, instead of a paste-like texture, a recovered soft and creamy self-supporting gel structure was observed when using DWPS. The ability to generate a range of textures in WPI gelation-based foods by using DWPS under different ionic conditions, is a feasible strategy for formulating high-protein foods for dysphagia—aimed to be either thickened fluids or soft solids. Additionally, this acquired knowledge is also relevant when formulating food gels for 3-D printing.

## 1. Introduction

Whey protein, obtained from the milk serum by-product generated during the cheese-making process, has been used extensively by the food industry as a versatile ingredient for its functional (gelling, foaming, emulsifying) and nutritional (high in essential amino acids) properties. The common food applications of whey protein include high-protein beverages, infant foods, confectionery, ice cream, spreads, processed meats, and bakery goods [1]. Whey protein is composed mainly of β-lactoglobulin (50–55%) and α-lactalbumin (20–25%), while the majority of the remaining proteins are made up of bovine serum albumin (BSA) and immunoglobulins [2]. Thermal gelation of whey protein occurs through initial denaturation/unfolding of protein followed by irreversible aggregation—with gel formation occurring in the presence of sufficient amount of protein, enough to form a stable network. The aggregation and gel properties of whey protein depend heavily on its major protein, β-lactoglobulin [3], where the aggregation is the result of non-covalent (i.e., hydrophobic, electrostatic, and steric forces) and covalent (formation of di-sulphide linkages between unfolded protein molecules) interactions. This is strongly affected by the ionic strength at which the gels are formed [4,5]. At low ionic strength and pH away from the isoelectric point of whey (pI~5.1), formation of a clear gel with a fine-stranded network (individual protein molecules ~3–5 nm) will be observed due to favourable conditions of electrostatic repulsion/stabilisation of whey proteins. In contrast, an opaque particulate gel is formed at high ionic strength [6]. The particulate gel has a coarse-stranded network with diameters of spherical aggregates ranging between 0.1 and 1 μm [7]. Mechanical measurements have shown that particulate gels are stiffer and less elastic than fine-stranded gels [8].

Studies have shown that native and modified starches can be used to alter the mechanical properties and microstructure of whey protein isolate (WPI) gels [9,10,11,12,13,14]. In these systems, the starch acted as a filler in the WPI network by contributing to an enhanced and denser final gel network [13] or promoted a weakened structure caused by flaws (prone to fracture) in the microstructure introduced by gelatinised starch [13,14,15]. Such manipulation of gel structures through the use of a filler has also been studied in WPI + cellulose nanocrystals [16] and meat myofibrillar protein + regenerated cellulose [17], where cellulose acted as a filler to create a more compacted network with higher gel strength. In our previous study, we modified waxy potato starch physically by high temperature treatment (from 120 to 150 °C) under constant shear, where starch granules were completely gelatinised and the resulting granules were reduced into fragments and their polymer chains. These treated starch samples are denoted as de-structured waxy potato starch (DWPS). These DWPS samples exhibited a wide range of molar masses and rheological properties (i.e., Newtonian, shear-thinning, shear-thickening, and anti-thixotropy behaviours) depending on their temperature treatment [15]. The DWPS containing smaller fragments and polymer chains can potentially create new binary WPI + starch gel structures that are different from those that contain conventional gelatinised starch. We observed that through controlling the ionic strength, DWPS can be used together with other food ingredients such as whey proteins to structure “clean-label” foods with desirable textural attributes. Of special interest is to produce textures that tackle the problem of dysphagia, a medical condition that hinders normal swallowing. Due to the risk of choking or aspiration, foods with soft texture and thickened fluids are used for dysphagia management to slow down the swallowing process, minimise chewing, and to protect the airway [18].

Currently, there is no literature reporting on the interaction between DWPS and whey protein isolate (WPI). Moreover, limited research has been conducted on the effect of salt on WPI + starch gels. Thus, in this study, we aim to elucidate the influence of salt on the interaction between WPI and DWPS by characterising the mechanical and microstructural properties of heat-induced WPI + DWPS composite gels at various ionic strengths (0–500 mM NaCl and CaCl_2_). Maltodextrin and gelatinised starch will also be used as controls. The results from this interaction study are essential for predicting the outcome when formulating high-protein foods for dysphagia as well as for food gel design in 3D printing.

## 2. Materials and Methods

### 2.1. Preparation of De-Structured Waxy Potato Starch 

DWPS sample was prepared according to the method by Ang, Matia-Merino, Lim and Goh [15] at 5% *w*/*w* waxy potato starch (Eliane 100, Avebe, Veendam, Netherlands) concentration with Milli-Q water. The sample was treated at 140 °C for 30 min under constant shear at 300 rpm with a turbine impeller. The sample was cooled to 20 °C and centrifuged at 28,804× *g* for 2 h. The supernatants were subsequently freeze-dried (BenchTop Pro with Omnitronics, SP Scientific, Suffolk, England). The molar mass and zeta-potential of the waxy potato starch and DWPS, and molar mass of maltodextrin of DE 2 (Glucidex-2, Roquettes Frères, Lestrem, France) used in the subsequent experiments have been reported previously (Table 1).

### 2.2. Preparation of Whey Protein + Starch Mixtures

Stock solutions of 25% *w*/*w* WPI (SureProtein^TM^ WPI 895, Fonterra Co-operative Group Limited, Auckland, New Zealand) and 11% *w*/*w* of DWPS were dispersed in Milli-Q water. Stock WPI solution was hydrated overnight under constant stirring at 4 °C. The DWPS stock solution was hydrated in a boiling water bath for 1 h, while being mixed with a vortex to ensure sample homogeneity. For comparison purposes, two additional samples (11% *w*/*w*) were used as control samples against DWPS: (i) gelatinised waxy potato starch heated at 95 °C for 30 min under constant shear at 300 rpm and (ii) maltodextrin heated in boiling water bath for 1 h. Mixtures containing maltodextrin or gelatinised starch served as control samples, as the former represents a system with similar total solids as in the mixed systems, and the latter is the control using unmodified starch needed to investigate the influence of DWPS on these mixtures. All the stock solutions were degassed (under vacuum) for 2 h and equilibrated to room temperature prior to use.

Stock NaCl solution (5 M) was added to stock solutions to produce 13% *w*/*w* WPI solution and mixtures (13% *w*/*w* WPI + 4% *w*/*w* maltodextrin or gelatinised starch or DWPS at varied NaCl concentrations of 0–500 mM). The concentrations of WPI and DWPS were selected based on preliminary trials. The same procedure was repeated with stock CaCl_2_ solutions (0.1 or 5 M) to obtain 13% *w*/*w* WPI and mixtures (13% *w*/*w* WPI + 4% *w*/*w* carbohydrate) at varied CaCl_2_ concentrations (0–500 mM). The pH values of pure WPI and mixed systems were ~6.5–6.7 and ~6.3–6.7 at varied NaCl and CaCl_2_ concentrations, respectively.

### 2.3. Phase Stability

The phase stability of pure WPI solutions and mixtures at various ionic strengths was determined visually (i.e., signs of phase separation or formation of two layers) over 24 h at 20 °C. Sodium azide (0.02% *w*/*w*) was added as preservative. 

### 2.4. Rheological Measurements

Rheological measurements were performed using a Paar Physica MCR 302 rheometer in controlled shear rate (CSR) mode (Anton-Paar, Graz, Austria) with a 25 mm diameter serrated plate geometry (PP25/2) and a plate Peltier temperature device (P-PTD 200/56/I) at 1 mm sample gap. Approximately 0.8 mL of pure WPI solution or mixture of WPI + gelatinised starch or maltodextrin or DWPS was loaded onto a geometry, pre-sheared at 10 s^−1^ for 60 s and rested for 5 min at 20.0 ± 0.1 °C. A thin layer of mineral oil around the sample and solvent trap were used during measurement to minimise evaporation during heating. Heat-induced gelation was done at 5 °C/min from 20–95 °C. The sample was then cooled to 20 °C at 5 °C/min. The heating and cooling cycles were conducted at 1% strain and 1 Hz frequency (within the linear viscoelastic region). The sample was then allowed to rest for 5 min at 20 °C before a frequency sweep was conducted from 0.1–20 Hz at 1% strain. *G*′ (storage modulus), *G*″ (loss modulus), G* (complex modulus), and *tan δ* (damping factor) were the viscoelastic parameters collected during testing. Each set of experiments was repeated three times with at least two measurements.

### 2.5. Zeta-Potential Measurements

The zeta-potential of maltodextrin (at its native pH) was determined using Zetasizer Nano ZS (Malvern Instruments Ltd., Malvern, UK) via electrophoresis and laser Doppler velocimetry techniques at sample concentrations of 0.5–2.0% *w*/*v*. The samples were measured in universal folded capillary cells (DTS1060C; Malvern Instruments Ltd., Malvern, UK) at 20 ± 0.02 °C. All experiments were repeated three times, each with five measurements.

### 2.6. Textural Measurements

#### 2.6.1. Gel Preparation

The pure WPI solutions and mixtures (~5 g) as described in Section 2.2 were loaded into a round silicone mould (20 mm diameter, 15 mm height). Noted that all samples were gently stirred before loading to minimise phase separation. Samples were then placed in a 90 °C water bath for 30 min to induce gelation. The gels were stored overnight at 20 °C before removal for further analysis.

#### 2.6.2. Compression Test

The hardness of the prepared gel samples was determined using a TA.XT plus texture analyser (Stable Micro System, Godalming, England). Compression test was done using a 35 mm cylindrical metal probe and a 50 kg load cell. The pre-test, test, and post-test speeds were set at 0.5, 1, and 5 mm/s, respectively. A trigger force of 0.049 N and 75% deformation were used for the measurement. The resulting plots of force versus time were used to determine the textural hardness as a maximum force during the compression. Each set of experiments was repeated three times with at least five measurements.

### 2.7. Microscopy Analysis

The effects of salts on the microstructure of gels were analysed via scanning electron microscopy (SEM) and confocal scanning laser microscopy (CSLM). Note that SEM was only carried out on the self-supporting gels (not paste-like samples).

#### 2.7.1. Scanning Electron Microscopy

The prepared gel samples were cut into small pieces (~3 mm) and soaked in a 0.1 M phosphate buffer containing 3% *w*/*v* glutaraldehyde and 2% *w*/*v* formaldehyde (pH 7.2) for 24 h at room temperature. The samples were washed three times in a 0.1 M phosphate buffer (pH 7.2) for 10 min, followed by dehydration using a series of ethanol solutions at increasing concentrations, i.e., 25, 50, 75, and 95% for 10 min each and at 100% for 1 h. Critical-point drying was carried out using liquid carbon dioxide and 100% ethanol with samples placed in a Polaron E3000 series II apparatus (Quorum, East Sussex, UK). Samples were then fractured, mounted on the aluminium stubs using double-sided tape samples, and sputter-coated with ~100 nm of gold (Bal-Tec SVD050, Los Angeles, CA, USA). The microstructure (2500× magnification) was taken using Quanta 200 Environmental scanning electron microscope (FEI Co., Hillsboro, OR, USA) at an accelerating voltage of ~10–15 kV.

#### 2.7.2. Confocal Scanning Laser Microscopy 

Samples prepared as described in Section 2.2 were loaded into laboratory-made welled slides and gelation was carried out at 90 °C for 30 min. The gels were stored at room temperature for 6 h before ~5 μL of Fast green dye (0.2% *w*/*v*) was added. The samples were then stored overnight at 20 °C, to allow a good dye penetration. Micrographs were taken using a confocal scanning laser microscope (Zeiss LSM900, Carl Zeiss AG, Jena, Germany), with a 63 × N.A, 1.4 oil immersion objective, and a helium/neon laser to excite the dye at 633 nm to detect protein with emission collected between 650–700 nm. Images were scanned twice at 5–10 μm below the coverslip, which were averaged to minimise noise. Each set of experiments was repeated twice.

### 2.8. Statistical Analysis

One-way analysis of variance with Tukey’s test was used to test significant differences among mean values at 95% confidence level using Minitab software (Minitab 18, Minitab Inc, Sydney, Australia).

## 3. Results and Discussion

### 3.1. Effect of Ionic Strength on Phase Stability

**Figure 1:**
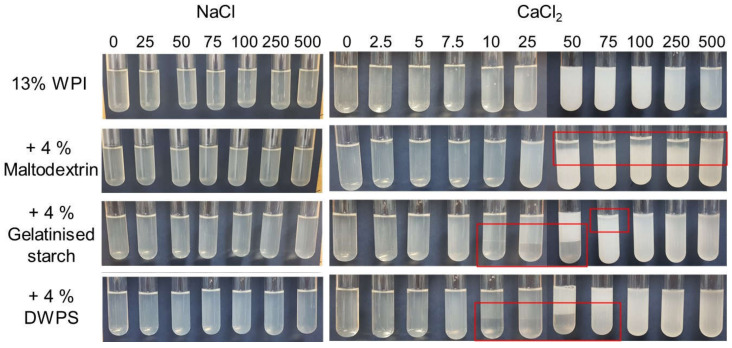
Effect of NaCl (0, 25, 50, 75, 100, 250, and 500 mM) and CaCl_2_ (0, 2.5, 5, 7.5, 10, 25, 50, 75, 100, 250, and 500 mM) on the phase stability of 13% *w*/*w* whey protein isolate (WPI) solutions and mixtures of 13% *w*/*w* WPI + 4% *w*/*w* maltodextrin or gelatinised starch or de-structured waxy potato starch (DWPS) after 24 h of storage at 20 °C.

According to the New Zealand Nutrition Foundation [20], foods can be categorised into low-, medium- and high-salt foods, which contain < 120, 120–600, and > 600 mg sodium per 100 g of food, which translates into NaCl concentrations of approximately < 50, 50–260, and > 260 mM. On the other hand, calcium is one of the most important minerals for the human body, and the recommended dietary allowance (RDA) for calcium is ~1000 mg for adult. Dairy products such as yoghurt are a source of calcium, containing 195 mg calcium per serving (150 g, ~33 mM) [21]. However, one serving of yoghurt only supplies ~20% of the daily RDA for calcium, making calcium-fortified foods the effective alternative to increase calcium intake. The effect of salts was evaluated in this study at the relevant concentrations between 0 and 500 mM. The phase stability of 13% WPI and 13% WPI + 4% maltodextrin or gelatinised starch or DWPS with the addition of NaCl or CaCl_2_ (0–500 mM) after 24 h at 20 °C is presented in Figure 1. Samples with added NaCl did not show any visual phase separation (Figure 1). Similarly, no phase separation was noted in pure WPI solutions at CaCl_2_ concentrations between 0 and 500 mM. However, increasing visual turbidity was observed at increasing CaCl_2_ concentrations between 10 and 100 mM. The results are in agreement with previous studies, where the higher ionic strength caused the formation of larger protein aggregates that scatter the light [22]. The subsequent increase in CaCl_2_ above 100 mM resulted in a decrease in sample turbidity (without sediment being detected). The decrease in turbidity is likely due to the dissociation effect contributed by the excess of chloride ions in the system. Some of the aggregated proteins cross-linked via calcium ions could dissociate in the presence of an increasing amount of counter-ions, which affect the electrostatic interactions, hence, lowering the turbidity of the samples. Such observations have been made in 7S and 11S soy protein solutions with CaCl_2_ and MgCl_2_, where a decrease in turbidity was observed after maximum divalent salt concentrations at ~30–40 mM [23].

In contrast to NaCl systems, visual phase separation was noted in mixed systems between 50 and 500 mM CaCl_2_ for WPI + maltodextrin, and between 10 and 75 mM CaCl_2_ for WPI + gelatinised starch or DWPS systems. The observed phase separation in the presence of calcium, could be explained by the unfavourable protein conformational changes induced by the divalent calcium ions due to bridging effects [24]. Moreover, the phase separation occurring at lower CaCl_2_ concentrations in protein + starch mixtures could be attributed to the gelatinised starch and DWPS being larger molecules than maltodextrin, which better facilitated the thermodynamic incompatibility (higher Gibbs free energy value) leading to separation [24,25]. This also led to a difference in the location of the polysaccharide-rich phase in the phase-separated samples—at the bottom (in the presence of the starches) or at the top (in the presence of maltodextrin). 

At concentrations ≥ 100 mM CaCl_2_, only single-phase systems were observed in the mixtures that contained gelatinised starch or DWPS. Given that the gelatinised starch and whey proteins are all initially negatively charged, the single-phase system may be the result of an optimum balance between attractive and repulsion forces among proteins and starch polymers at high CaCl_2_ concentrations, which results in thermodynamic compatibility. It could also be related to the dissociative effect on proteins as described above, contributed by high concentrations of chloride ions, which reverse the shielding and bridging effects of calcium ions resulting in some of the protein molecules regaining their negative charges and getting dissociated, decreasing the incompatibility with the polysaccharide fraction [24,25]. 

### 3.2. Effect of Ionic Strength on Gelation Temperature

**Figure 2:**
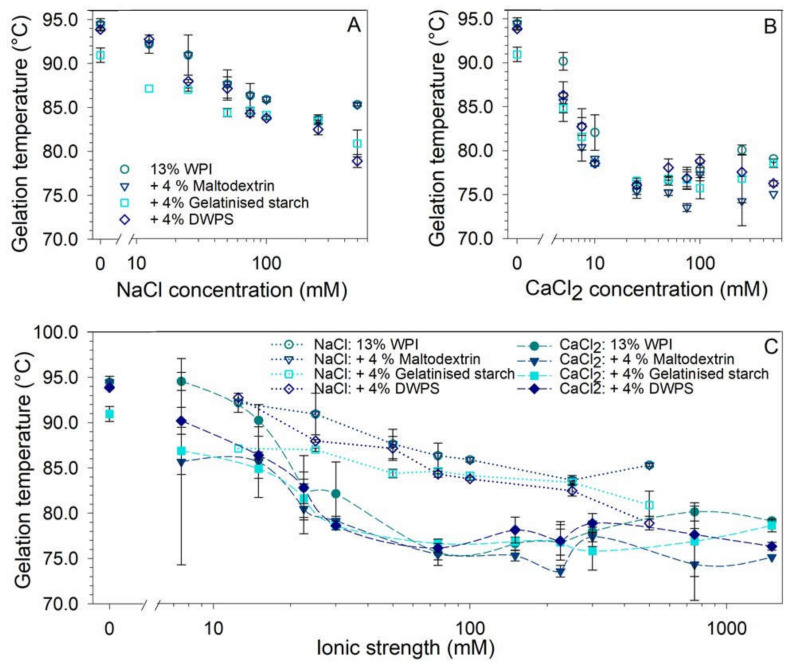
Effect of salts on the gelation temperature of 13% *w*/*w* WPI and 13% *w*/*w* WPI + 4% *w*/*w* maltodextrin or gelatinised starch or DWPS at varying: (**A**) NaCl concentrations of 0, 12.5, 25, 50, 75, 100, 250, and 500 mM, and (**B**) CaCl_2_ concentrations of 0, 2.5, 5, 7.5, 10, 25, 50, 75, 100, 250, and 500 mM, and (**C**) effect of NaCl and CaCl_2_ on gelation temperature at equivalent ionic strengths (lines serve as visual aids), with an inset graph showing added ionic strength from 0 to 50 mM. The gelation temperatures were obtained from temperature sweep when storage modulus (*G*′) crossed over loss modulus (*G*″) during the heating phase at 1% strain and 1 Hz frequency, values are plotted as means ± standard error.

The individual effects of NaCl (0–500 mM) and CaCl_2_ (0–500 mM) on the gelation temperature of 13% *w*/*w* WPI and mixed systems are presented in Figure 2A,B, respectively, as well as the plot against the calculated ionic strength (0–1500 mM) in Figure 2C. The gelation temperature of WPI was not significantly affected by the addition of maltodextrin or DWPS, whereas gelatinised starch significantly decreased the gelation temperature from 95 to 91 °C (Figure 2A). Such observation could be due to gelatinised starch being a larger and more negatively charged molecule (Table 1), which is better at promoting segregative interactions between starch and protein molecules, causing enhanced protein denaturation and aggregation [26,27]. The addition of small quantities of both NaCl and CaCl_2_ rapidly reduced the gelation temperature of the system by ~10–15 °C (Figure 2A,B). The reduction plateaued off at 75 mM NaCl (Figure 2A) and 25 mM CaCl_2_ (Figure 2B), which corresponds to a similar ionic strength of ~75 mM NaCl (Figure 2C). However, CaCl_2_ was clearly more effective than NaCl at reducing gelation temperature (Figure 2C). A similar observation was made by Puyol, Pérez, and Horne [28], where the increase in salt concentrations resulted in a reduction in the gelation temperature of WPI. Such reduction can be attributed to enhanced aggregation contributed by the shielding of charged protein molecules by cations and intra- and inter-molecular bridges formed by calcium ions between negatively charged protein molecules [29,30]. In conclusion, above 75 mM total ionic strength for any of the systems (NaCl or CaCl_2_), the gelation temperature of both pure WPI and mixed systems was no longer significantly affected by further salt addition. Hence, the results indicate that both salts, especially CaCl_2_, could be used to lower gelation temperatures to minimise energy consumption during gel formation.

### 3.3. Rheological, Textural, and Microstructural Properties

#### 3.3.1. Effect of NaCl

**Figure 3:**
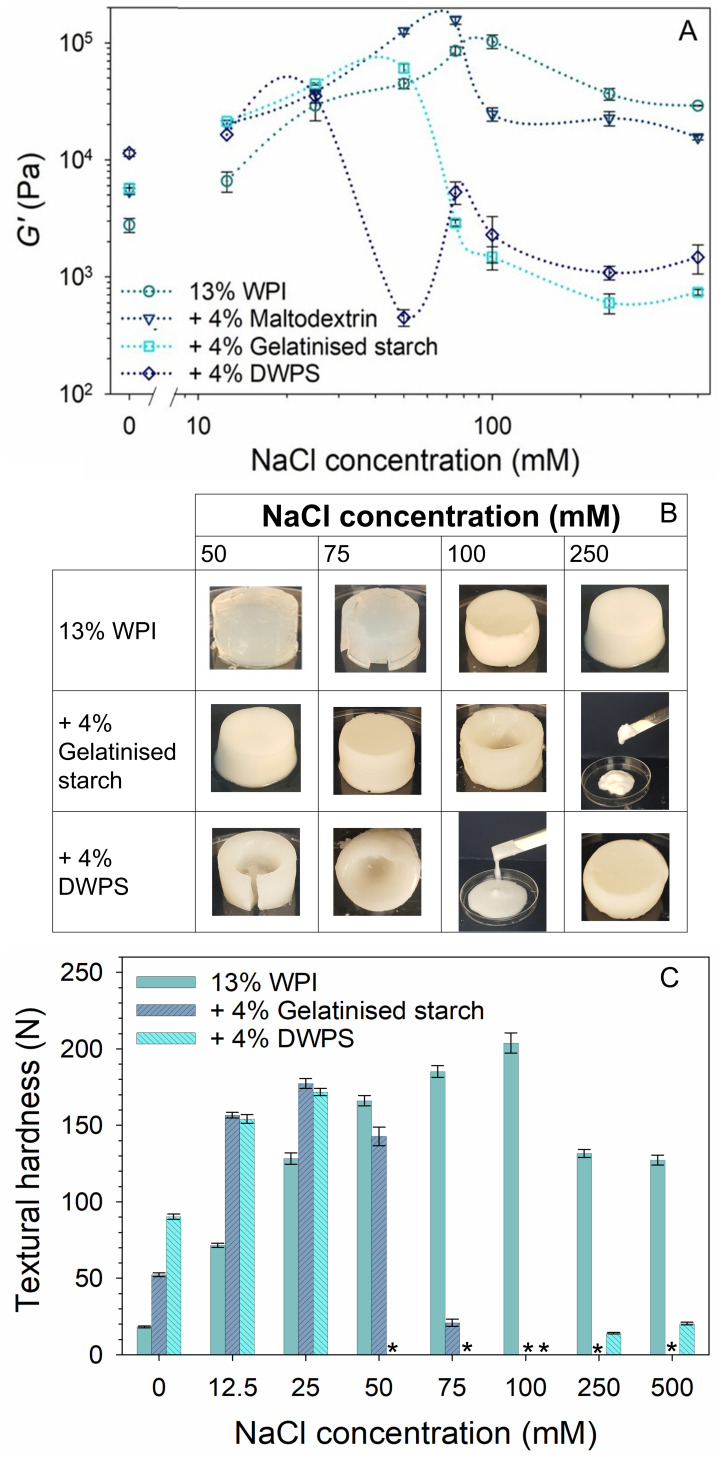
Effect of NaCl on the: (**A**) *G*′ from frequency sweep at 1% strain and 1 Hz frequency of 13% *w*/*w* WPI and 13% *w*/*w* WPI + 4% *w*/*w* maltodextrin, gelatinised starch, or DWPS with 0–500 mM NaCl at 20 °C. Lines in the graphs serve as visual aids, (**B**) visual appearance of 13% *w*/*w* WPI and 13% *w*/*w* WPI + 4% *w*/*w* gelatinised starch or DWPS at 50, 75, 100, and 250 mM NaCl, (**C**) textural hardness of 13% *w*/*w* WPI and 13% *w*/*w* WPI + 4% *w*/*w* gelatinised starch or DWPS with 0–500 mM NaCl at room temperature. Values are plotted as means ± standard error. Note that compression test was not performed on inhomogeneous/paste-like gels (denoted with *).

It is worth noting that all the samples in this study can be defined rheologically as gels, as they exhibited *G*′, above *G*″, and *tan δ* < 0.2. In addition, G* values were dominated by *G*′ (see Figure S1 in the Supplementary Materials) so only the *G*′ values will be used to compare and discuss results as it represents the gel strength of a network [31]. The effects of NaCl on *G*′ of pure WPI and composite gels are presented in Figure 3A. Pure WPI and WPI + maltodextrin showed similar increasing trends in *G*′ values with increasing NaCl concentrations up to 100 and 75 mM, respectively. Further increase in NaCl concentrations caused a slight reduction in *G*′ and the values plateaued off to ~36.8 and ~15.7 kPa, respectively, at 250 mM NaCl. Such behaviour of NaCl in WPI gels was also noted by Urbonaite et al. [7] when NaCl increased from 0 to 300 mM. The authors observed an increase in WPI gel stiffness where the network strands grew thicker up to an optimum NaCl concentration of 150 mM. Further increase in NaCl after this optimum concentration led to even thicker strands. The authors observed microstructures with coarser network (1.6–2.0 μm) with a lower protein connectivity when NaCl concentration was increased from 150 to 300 mM, which also led to a decrease in gel stiffness values from ~430 to ~80 kPa.

Without NaCl, WPI + DWPS exhibited a unique synergistic increase in *G*′ and hardness values (~11.4 kPa and ~90 N) that was almost twice the values of that WPI + gelatinised starch (~5.7 kPa and ~52 N). Such a significant increase in WPI gel strength has not been reported before. However, the unique synergistic effect of DWPS in WPI gel diminished with the addition of NaCl as similar *G*′ and hardness values were noted in among the mixed systems (i.e., WPI + maltodextrin or gelatinised starch or DWPS) at 12.5 mM NaCl (Figure 3A,C). Increased *G*′ values were noted in WPI + gelatinised starch and WPI + DWPS up to NaCl concentrations of 50 mM and 25 mM, respectively.

The further increase in NaCl concentrations for WPI + gelatinised starch (75 mM) and WPI + DWPS (50 mM) led to lower *G*′ values. With WPI + gelatinised starch gels, *G*′ plateaued off at ≥250 mM. In contrast, a recovery in *G*′ value was noted in WPI + DWPS at 75 mM, followed by a gradual decrease in *G*′ at 100 mM before plateauing at ≥ 250 mM.

**Figure 4:**
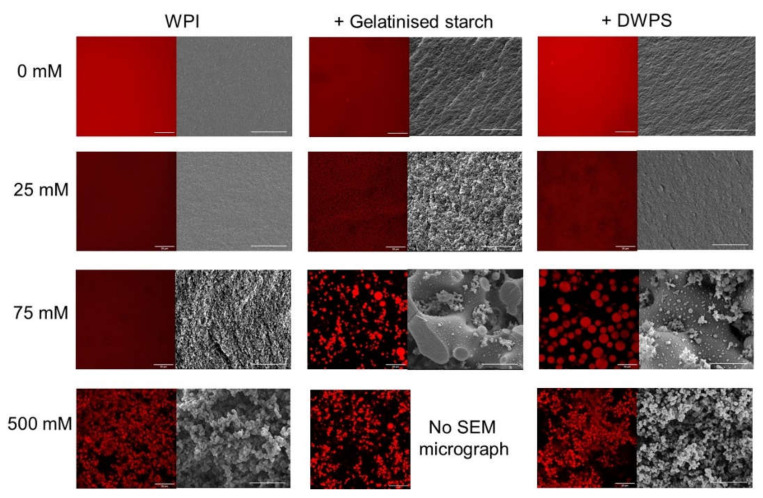
Effect of NaCl on the confocal scanning laser microscopy (CSLM) micrographs (630×) and scanning electron microscopy (SEM) micrographs (2500×) of 13% *w*/*w* WPI and 13% *w*/*w* WPI + 4% *w*/*w* gelatinised starch or DWPS gels at 0, 25, 75, and 500 mM NaCl. Note that SEM was not done on paste-like samples. The scale bars are 20 μm.

In order to further understand the texture and microstructure of these samples, gels of pure WPI and WPI + gelatinised starch or DWPS containing 0–500 mM NaCl were formed, where their textural attributes (consistency and homogeneity) were evaluated visually at macroscopic and microscopic scales (Figure 3B and Figure 4) and measured using a texture analyser to obtain the hardness values (Figure 3C). Self-supporting gels were obtained throughout the tested NaCl concentrations of 0–500 mM for pure WPI gels. As for WPI + gelatinised starch, gels with different textural properties were observed at increasing NaCl concentrations, including: (i) self-supporting gels, (ii) self-supporting gels with a liquid centre, and (iii) paste-like weak gels at ≤ 75, 100, ≥ 250 mM NaCl, respectively. WPI + DWPS samples exhibited an even more varied range of textural properties, including those observed in WPI + gelatinised starch as above (i, ii, and iii at ≤ 25, 50–75 and 100 mM NaCl concentrations, respectively) and an additional recovery of texture at ≥ 250 mM NaCl, where soft and creamy self-supporting gels were formed.

In general, *G*′, the visual properties of the samples (consistency and homogeneity), and the hardness values correlated well with each other. The decreased *G*′ values observed for WPI + gelatinised starch or DWPS (Figure 3A) could be explained by the inhomogeneity of the gels (Figure 3B), where self-supporting gels with a liquid centre were observed at NaCl concentrations mentioned above. A further increase of NaCl concentrations to ≥ 250 mM in WPI + gelatinised starch and 100 mM in WPI + DWPS gels resulted in samples having a paste-like weak gel consistency, hence the observation of lower *G*′ values compared to those at the lower NaCl concentrations. 

These differences in gel texture can be further explained by observing the microstructure of the composite gels (Figure 4). At a low NaCl concentration of 25 mM, the addition of gelatinised starch or DWPS to WPI seems to cause increased protein aggregation, which is evidenced by the increased roughness observed in the micrographs of composite gels as compared to that of a pure WPI sample. At 75 mM, the liquid portion was analysed using CSLM, whereas the gel portion was imaged with SEM. For the liquid portion, WPI existed as dispersed spherical droplets in the continuous phase (CSLM micrograph in Figure 4 with DWPS). Such microstructure is typical of micro-phase separation of incompatible biopolymers, where the adoption of such spherical conformation minimises the overall surface tension of the system [32]. On the other hand, the gel network observed by SEM at 75 mM was different from that observed at 25 mM NaCl, where the former appeared to be a more open network. The occurrence of thermodynamic incompatibility resulting in an inhomogeneous gel could be a plausible reason for the reduced *G*′ value of WPI + gelatinised starch or DWPS (Figure 3A). Such micro-phase separation has also been observed in 13% *w*/*w* WPI + 0.5% *w*/*w* carrageenan at 50–100 mM NaCl [33]. The increase of NaCl from 75 to 100 mM resulted in a change in texture from self-supporting gels with a liquid centre to a weak gel with paste-like consistency (Figure 3B). The paste-like texture is likely caused by thermodynamic incompatibility between WPI and DWPS at 100 mM NaCl, resulting in a microstructure resembling that of the liquid portion of WPI + DWPS at 75 mM (i.e., WPI unable to form a continuous network) [34].

At high NaCl concentrations (500 mM), the good protein connectivity seen in the micrographs of WPI + DWPS (Figure 4 with DWPS) suggests that the recovery of a self-supporting gel structure in WPI + DWPS was due to the dominating protein–protein interactions. Hence, such enhanced interactions were facilitated by high levels of NaCl. In contrast, poor protein connectivity was noted in WPI + gelatinised starch at 500 mM NaCl (Figure 4 with gelatinised starch). The poor protein connectivity could explain the lower *G*′ values and the paste-like consistency observed in WPI + gelatinised starch as compared to WPI + DWPS. As shown earlier, gelatinised starch is more negatively charged than DWPS (Table 1), which suggests that the former is likely to compete with proteins for sodium ions. The competition between protein and gelatinised starch for positive ions led to a lower amount of available sodium ions to neutralise the negative charges on protein molecules. Hence, the remaining negative charges on the proteins were able to cause protein–protein repulsions and prevented good connectivity. Another possible reason for the observed weaker structure in WPI + gelatinised starch could be that the gelatinised starch, being a larger molecule (Table 1), was more effective in disrupting the connectivity of the protein network.

#### 3.3.2. Effect of CaCl_2_

**Figure 5:**
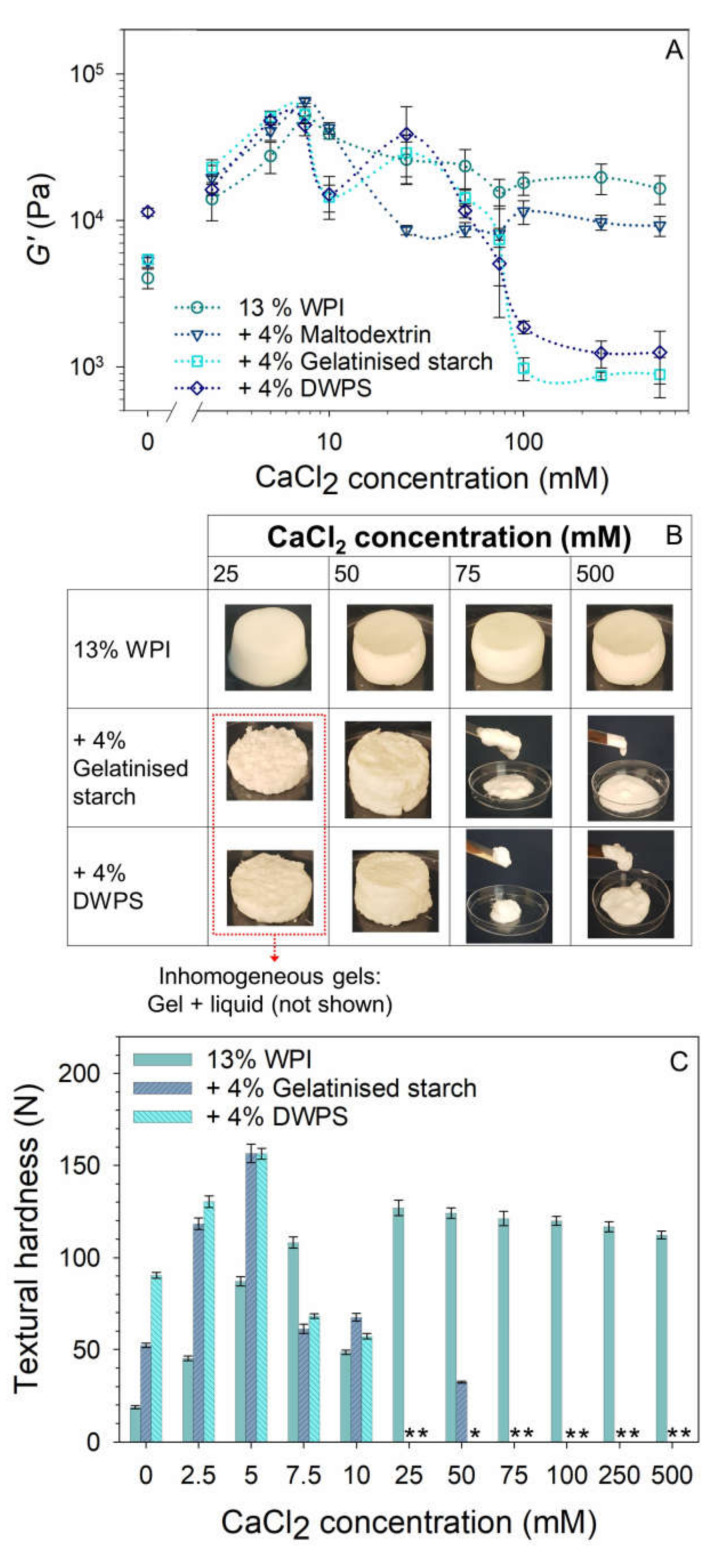
Effect of CaCl_2_ on the: (**A**) *G*′ from frequency sweep at 1% strain and 1 Hz frequency of 13% *w*/*w* WPI and 13% *w*/*w* WPI + 4% *w*/*w* maltodextrin, gelatinised starch or DWPS with 0–500 mM CaCl_2_ at 20 °C. Lines in the graphs serve as visual aids, (**B**) visual appearance of 13% *w*/*w* WPI and 13% *w*/*w* WPI + 4% *w*/*w* gelatinised starch or DWPS at 25, 50, 75 and 500 mM CaCl_2_, and (**C**) textural hardness of 13% *w*/*w* WPI and 13% *w*/*w* + 4% *w*/*w* gelatinised starch or DWPS with 0–500 mM CaCl_2_ at room temperature. Values are plotted as means ± standard error. Note that compressed test was not performed on inhomogeneous/paste-like gels (denoted with *).

**Figure 6:**
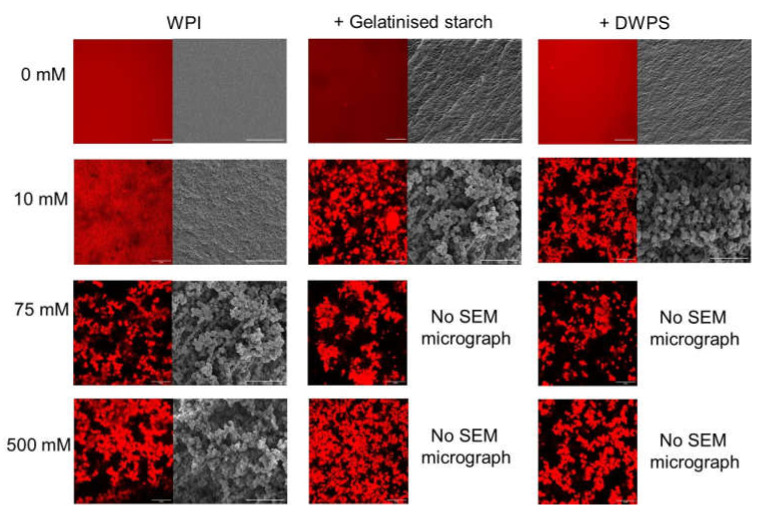
Effect of CaCl_2_ on the CSLM micrographs (630×) and SEM micrographs (2500×) of 13% *w*/*w* WPI and 13% *w*/*w* WPI + 4% *w*/*w* gelatinised starch or DWPS gels at 0, 10, 75, and 500 mM CaCl_2_. Note that SEM was not done for paste-like samples. The scale bars are 20 μm.

The effect of CaCl_2_ on the *G*′, visual appearance, and gel hardness of pure WPI and composite gels is presented in Figure 5. Like NaCl, increasing trends were observed in both *G*′ and hardness values at the initial increase of CaCl_2_ concentrations (Figure 5A,C). However, the occurrence of maximum *G*′ and hardness values was observed at considerably lower concentrations in the presence of calcium (~5–7.5 mM CaCl_2_ versus ~25 mM NaCl for WPI + gelatinised starch or DWPS). These observations could again be attributed to the ability of calcium ions to form bridges between protein molecules, which facilitated protein aggregation [29,30]. At ≤ 10 mM CaCl_2_, all the gels were self-supporting with good protein connectivity as observed in both the CSLM and SEM micrographs (Figure 6). However, at 25 mM, inhomogeneous gels were noted for WPI + gelatinised starch or DWPS (Figure 5B), which were attributed to the phase separation between WPI and starch polymers (Figure 1) occurring during gel formation. These two opposing forces present during gelation—segregative and associative—are likely to result in heterogeneous gels [35]. At 75 mM, gelatinised starch or DWPS in the composite gels disrupted the connectivity of protein network (Figure 6), resulting in the formation of a weak gel with paste-like consistency (Figure 5B). Even though increased protein connectivity was observed in CSLM micrographs of WPI + gelatinised starch and WPI + DWPS at 500 mM CaCl_2_ (Figure 6), the gels still retained their paste-like consistency (Figure 5B). Our observations were different from the study conducted by Yang, Luan, Ashton, Gorczyca, and Kasapis [36], where the authors noted self-supporting composite gels containing 15% WPI + 3–5% wheat starch at CaCl_2_ concentration from 5–192 mM. In their system, starch existed as a filler that was trapped in the protein continuous network. The differences in gel properties could be due to the differences in sample preparation. In our experiments, starch gelatinisation was carried out under shear before mixing with WPI stock solution, followed by the heating of this mixture, whereas in Yang et al. [36], the starch was gelatinised during the heat-induced gelation of the composite gel. It is likely that the starch in Yang et al. [36] was less gelatinised and had some of its granular structure, which led to the entrapment of starch in the protein network. Consequently, their gelatinised starch might not be as effective in disrupting the connectivity of the protein network at high CaCl_2_ concentrations as compared to the inclusion of gelatinised starch or DWPS in our gels. In addition, a higher protein concentration (15% *w*/*w* protein) was also used in their system as compared to ours (13% *w*/*w* protein), which could result in a gel having prominent protein gel characteristics with a stronger network.

### 3.4. General Remarks

The above findings have demonstrated that both NaCl and CaCl_2_ can be used to manipulate the microstructure and mechanical properties of composite systems based on heat-induced whey protein gels with added starch—such knowledge is valuable in food formulations. In particular, the WPI + DWPS system was able to yield a plethora of gel textures with desirable attributes at varying NaCl concentrations that can be used in different food applications. The range of possible textures with WPI + DWPS gels—such as paste-like texture (100 mM NaCl or 75–500 mM CaCl_2_) and soft self-supporting gels (250–500 mM NaCl)—can be utilised in 3D printed foods as well as when formulating pH neutral high-protein-dense foods with or without calcium fortification for the growing senior population and dysphagia sufferers. However, future work is recommended to evaluate the feasibility of textural manipulation using DWPS in combination with ionic strength variations, for specific high-protein food matrices.

## 4. Conclusions

Visual macro-phase separation was only observed in mixtures containing CaCl_2_ (not NaCl) and the separations were observed at 50–500 mM in WPI + maltodextrin and 10–75 mM in WPI + gelatinised starch or DWPS systems. By controlling the NaCl concentration in WPI + DWPS systems—affecting micro-phase separation and/or protein connectivity—we obtained a plethora of gel textures suitable for different food applications. Such gel textures include homogeneous strong self-supporting gels, inhomogeneous self-supporting gels with a liquid centre, and weak gels with paste-like consistency. Recovery of self-supporting gel structures was also noted at ≥ 250 mM NaCl, where gels exhibited a soft and creamy texture. Weak gels with paste-like consistency were also noted with WPI + starch systems containing 75–500 mM CaCl_2_. These systems can serve as a form of mineral carrier for calcium-fortified foods. The ability to generate a range of textures in WPI gelation-based foods by using DWPS under different ionic conditions is a feasible strategy for formulating high-protein foods (with or without calcium fortification) for 3D printing as well as for dysphagia sufferers, where the range of food textures include thickened fluids and soft solids.

## Figures and Tables

**Table 1:** Molar mass and zeta-potential of maltodextrin, waxy potato starch, and de-structured waxy potato starch (DWPS).

	Molar Mass (Da)	Zeta-Potential (mV)
Maltodextrin	5.89 × 10^3^	−8.5 ± 0.2 ^b^
Waxy potato starch	3.70 ± 0.16 × 10^8 a^	−31.0 ± 0.5 ^a^
DWPS	1.57 ± 0.04 × 10^6 b^	−2.8 ± 0.3 ^c^

Values are expressed as means ± standard error. Values in the same column denoted with the same superscripts are not significantly different (*p* ≤ 0.05). Note that the molar mass and zeta-potential of waxy potato starch and DWPS are obtained from previously reported data [15], and the molar mass of maltodextrin is referenced from Castro et al. [19] whereas the zeta-potential of maltodextrin was determined as described in Section 2.5. The zeta-potential values of the maltodextrin, waxy potato starch, and DWPS were determined at their native pHs.

## Data Availability

Not applicable.

## Supplementary Materials

The following supporting information can be downloaded at: https://www.mdpi.com/article/10.3390/gels8070399/s1, Figure S1: Frequency sweep of 13% *w*/*w* WPI + 4% *w*/*w* maltodextrin at 75 mM NaCl and 13% *w*/*w* WPI + 4% *w*/*w* 140 °C DWPS at 50, 100, and 500 mM NaCl at 20 °C at 1% strain. Values are plotted as means ± standard error.
