# Supplementary material for: High-Protein Foods for Dysphagia: Manipulation of Mechanical and Microstructural Properties of Whey Protein Gels Using De-Structured Starch and Salts
Source: Gels. 2022 Jun 23;8(7):399. doi: 10.3390/gels8070399 (PMC9318909; doi:10.3390/gels8070399)
Supplement: Supplementary file 1 [file gels-08-00399-s001.zip › gels-1755094-supplementary.pdf]

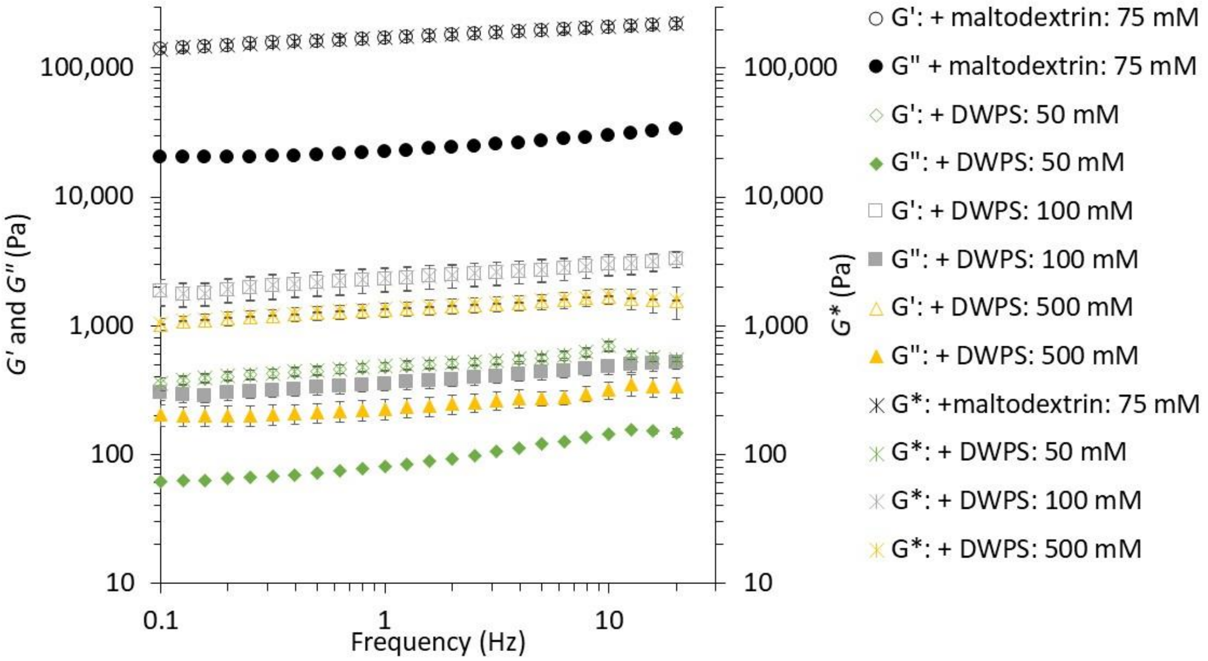

Figure S1: Frequency sweep of 13% w/w WPI + 4% w/w maltodextrin at 75 mM NaCl and 13% w/w WPI + 4% w/w 140 °C DWPS at 50, 100, and 500 mM NaCl at 20 °C at 1% strain. Values are plotted as means ± standard error.
